# Designing Effective Multidisciplinary Team Meetings in the Greek National Health System: Opinions, Obstacles, and Implementation

**DOI:** 10.7759/cureus.80550

**Published:** 2025-03-13

**Authors:** Iliana Papadopoulou, Nikolaos Benetatos, Konstantina Soultana Kitsou, Dimitra Latsou, Maria Saridi, Aikaterini Toska

**Affiliations:** 1 Department of Surgery, General University Hospital of Patras, Patras, GRC; 2 Department of Public Administration, University of Neapolis, Paphos, CYP; 3 Department of Nursing, University of Thessaly, Volos, GRC; 4 Department of Epidemiology and Public Health, University of Thessaly, Volos, GRC

**Keywords:** health professionals, health system, mdtm protocols, multidisciplinary team meetings (mdtm), therapeutic plan

## Abstract

Introduction: The implementation and accessibility of multidisciplinary team meetings (MDTMs), which ensure equal opportunities for health professionals to participate, are fundamental to the functioning of any national health system.

Aim: This study aims to describe the main characteristics of MDTMs and assess the opinions and experiences of healthcare professionals who participated in these meetings.

Materials and methods: We conducted a cross-sectional study of health professionals who participated in MDTMs at tertiary referral health centers within the Greek national health system. We employed a simple random sampling methodology, and the questionnaires were completed automatically. The survey was conducted from February to April 2022.

Results: Ninety-eight questionnaires were distributed, and 72 complete responses were returned (response rate: 73.5%). The mean age of respondents was 47.2 ± 7.4 years. Sixty-five participants (90.3%) were specialized doctors, and thirty-nine (54.17%) stated that the time allocated for MDTM sessions was insufficient. Meanwhile, the majority agreed that MDTMs improved patient treatment allocation (n=59, 82%), were beneficial for healthcare professionals’ education (n=60, 83.4%), and enhanced the practical and educational skills of medical interns (n=57, 79.2%). Regarding case discussions in MDTMs, participants agreed that comorbidities (n=67, 93.1%), social-psychological factors (n=59, 82%), and patients’ preferences (n=62, 86.1%) should always be considered during meetings. The primary challenges in achieving a complete therapeutic plan were identified as inadequate teamwork (n=11, 15.3%), complexity of referred cases (n=9, 12.5%), and time constraints (n=9, 12.5%).

Conclusions: Weaknesses in the healthcare system have emerged, necessitating action to address areas requiring improvement. Differences in the conceptual framework of MDTM functionality, particularly regarding specialists' specific roles and responsibilities, have been observed. Since the Greek national health system lacks specific protocols for the universal implementation of MDTMs, this study underscores the need for structured MDTM protocols in Greece to enhance healthcare professionals’ participation and optimize patient care.

## Introduction

Multidisciplinary team meetings (MDTMs) have been implemented globally to enhance the care of cancer patients and individuals with various healthcare needs. These meetings bring together healthcare professionals from multiple disciplines, equipping them with the necessary skills to provide comprehensive care. They promote coordination between specialists and communication with patients, thereby improving the decision-making process for treatment and enhancing the quality of care [1-4].

Regardless of their role, MDTMs involve specialists and anyone involved in delivering healthcare services. These meetings are central to the patient care pathway, where various sources integrate clinical information. The benefits of MDTMs include improved healthcare processes and adherence to up-to-date, evidence-based treatment recommendations for different types of cancer [5-8]. Additionally, MDTMs contribute to shorter case turnaround times, a greater emphasis on disease-related perspectives, skills development, clinical experience, training opportunities for new practitioners, and identifying patients suitable for clinical trials [8].

MDTMs are particularly valuable for complex cases, which account for 25% to 40% of cases reviewed [9,10]. However, the time available for discussing these cases during MDTMs may be insufficient due to the high volume of cases requiring treatment [11-13]. Poor participation, insufficient data, inadequate administrative support, and ineffective leadership within MDTMs can negatively impact decision-making. Furthermore, the potential effect of MDTMs on treatment plans should not be overlooked [14].

Although there is general agreement on the value of MDTMs, concerns remain regarding their structure due to the resource demands resulting from the increasing incidence of cancer cases, the involvement of multiple specialists, the high frequency of meetings for early treatment, the assessment based on improved diagnostic methods, and the complexity of treatment algorithms. Information on the structure and function of individual MDTMs is rarely reported in the literature, and compliance standards vary [15,16].

The establishment, coordination, and operation of MDTMs and equal access for healthcare professionals are key elements of a well-functioning healthcare system. This study was conducted in both teaching and non-teaching tertiary hospitals. To provide a basis for structured and targeted improvements in cancer care, we explored the views of healthcare professionals on MDTMs. Despite the proven benefits of MDTMs internationally, little is known about their implementation and challenges within the Greek healthcare system. To our knowledge, this is the first and only study of its kind in Greece.

This research aims to enhance our understanding of healthcare professionals' perceptions of MDTMs, explore the experiences of multidisciplinary team members, and gather information on resource allocation. The study seeks to present the characteristics of the meetings and the participating groups while outlining both advantages and drawbacks throughout the process. The findings aim to inform the development of standardized protocols that could be applied to other healthcare systems.

## Materials and methods

A cross-sectional survey was conducted to assess healthcare professionals' views on their participation in MDTMs within tertiary hospitals of the Greek national health system. We employed a random sampling approach to minimize selection bias across different specialties and institutions. Potential confounders, such as institutional differences in MDTM protocols, were addressed by stratifying responses based on hospital type (teaching vs. non-teaching). Despite efforts to ensure diversity, we acknowledge the potential overrepresentation of physicians compared to other healthcare professionals.

A simple random sampling method was used, and data were collected through self-administered online questionnaires completed by participating healthcare professionals. Inclusion criteria encompassed healthcare professionals actively involved in MDTMs, including physicians, nurses, and administrative staff. Exclusion criteria included professionals with less than six months of MDTM involvement and those who declined to provide informed consent. The survey period spanned from February to April 2022.

Survey sample

The data for the study were collected using a printed questionnaire table, which was distributed to healthcare professionals participating in the MDTMs of the second and sixth Health Regions of Greece, specifically at the General University Hospital of Patras and the General Anticancer Hospital of Piraeus "METAXA." A power calculation was conducted to determine an adequate sample size. Assuming a 95% confidence level, an expected effect size of 0.3, and an anticipated response distribution of 50%, the required sample size was approximately 70 participants to achieve 80% power in detecting meaningful associations. The study distributed 98 questionnaires, of which 72 were fully completed and included in the analysis, yielding a response rate of 73.5%.

Survey questionnaire

Translation

A structured questionnaire on the functioning of MDTMs and the views of their participants, including perceived benefits and barriers, was designed and evaluated by Rosell et al. [17]. The original questionnaire was in English (with licensed usage of the original questionnaire). Before the study, the authors translated the questionnaire into Greek. Subsequently, a bilingual academic with experience in the field translated it back into English. The bilingual academic and the authors then compared the two versions (forward and backward translation) and corrected any discrepancies in the Greek version. During the translation process, modifications were made to the questionnaire to ensure it corresponded appropriately to Greek clinical practice.

Pilot Survey

The translated questionnaire was given to seven healthcare professionals as a pilot test. Face validity was assessed by gathering the healthcare professionals' views on the tool's usefulness to determine whether it appeared to measure what it intended [18]. Subsequently, content validity was evaluated to assess whether the questions in the questionnaire adequately measured the concepts they were designed to explore [19]. The healthcare professionals provided feedback on the accuracy of the wording and the clarity of the questions. The authors considered their observations and made the necessary revisions to create the final version of the distributed questionnaire.

Questionnaire Description

The questionnaire consisted of three parts. The first part included nine questions related to the demographic and professional characteristics of the participants, their frequency of participation in MDTMs, and the time spent in MDTMs. Specifically, demographic characteristics encompassed data on age, gender, occupation (physician, nurse, secretary/coordinator), specialty (surgery, pathology, radiology, pathological anatomy/cytology), and the Regional Health Authorities (YPE) where the participants work (second, sixth YPE). Additionally, this section included questions about the number of MDTMs participants attended per week, the type of MDTMs (local, regional, national), the time spent each week in MDTMs, and their opinion on the adequacy of the time allocated to them. Surgical specialties included urology, thoracic surgery, neurosurgery, and gynecology, while medical specialties encompassed hematology, pulmonology, neurology, oncology, and gastroenterology. Radiology also includes nuclear medicine.

The second part comprised questions related to the purpose and implementation of MDTMs. Participants were asked to select and rank the most important reasons for conducting MDTMs from 14 suggested options and to rate three suggestions related to their role in MDTMs. Regarding their role, participants expressed their views using a 7-point Likert scale, ranging from 1 (strongly disagree) to 7 (strongly agree), with the option to select “do not know/does not concern me.”

The third part focused on the structure and guidelines of MDTMs. Specifically, participants were asked to rate 11 statements regarding their views on the benefits, structure, and guidelines related to MDTMs and six statements regarding their perspectives on the patients discussed in MDTMs. Finally, to identify the advantages or obstacles to reaching a common treatment plan, participants were asked to select and rank five out of the 14 most significant advantages of MDTMs and five out of the 16 most important disadvantages. The full questionnaire is provided as a Supplementary File accompanying this manuscript to ensure transparency.

Ethical considerations

The study was approved by the Ethics, Research, and Deontology Committee of the General University Hospital of Patras (Ref. No. 64/15-02-2022) and the Board of Directors of the General Anticancer Hospital of Piraeus "METAXA" (Ref. No. 6/11-03-2022). Informed consent was obtained from all participants, and data confidentiality was ensured in accordance with institutional guidelines.

Statistical methods

Data were analyzed using the statistical package SPSS Statistics version 25 (IBM Corp. Released 2017. IBM SPSS Statistics for Windows, Version 25.0. Armonk, NY: IBM Corp.). Descriptive statistics were used to describe the sample and are presented in absolute numbers and percentages. Variables were ordered, and non-parametric tests were selected. To compare variables with two categories, the Mann-Whitney test was applied, while for the comparison of variables with three or more categories, the Kruskal-Wallis test was used. Categorical variables were compared using Pearson's chi-squared (χ²) test. A significance level of <0.05 was used in all cases.

## Results

Demographic and occupational characteristics

The mean age of the participants was 47.2 ± 7.4 years. A total of 41 participants (56.9%) belonged to the age group >45 years, and 38 (52.8%) were male. Additionally, 65 (90.3%) participants worked as medical specialists, with 34 (47.2%) being internists and 22 (30.6%) surgeons. Furthermore, 40 (55.6%) participants worked in a hospital under the second YPE. Table 1 presents the demographic and professional characteristics of the study participants.

**Table 1:** Demographic and occupational characteristics YPE: Regional Health Authority

	Number	Percentage %
Age		
<45 years old	31	43.1
>45 years old	41	56.9
Sex		
Male	38	52.8
Female	34	47.2
Occupation		
Resident physician	2	2.8
Specialized physician	65	90.3
Nurse	3	4.2
Secretary/coordinator	2	2.8
Specialty		
Surgery (including urology, orthopedics, thoracic surgery, gynecology, and neurosurgery)	22	30.6
Internal medicine (including pulmonology, hematology, oncology, gastroenterology, and neurology)	34	47.2
Radiology (including nuclear medicine)	8	11.1
Pathology/cytology	3	4.2
None of the above	5	6.9
YPE		
2^nd^ YPE	40	55.6
6^th^ YPE	32	44.4

Of the participants, 48 (66.7%) attended fewer than one MDTM per week, with meetings held only at the local level rather than at the regional or national level. Additionally, 35 (48.6%) prepared for one hour before participating in MDTMs, while 24 (33.3%) prepared for only half an hour (mean: 1.11 ± 1 hour). The mean participation time was 0.9 ± 0.2 hours. After MDTMs, 27 (37.5%) worked for approximately one hour, while 35 (48.6%) worked for about half an hour, with a mean post-meeting work time of 0.6 ± 0.4 hours.

Insufficient time allocation was reported by 39 (54.17%) participants, highlighting the need for administrative restructuring to support more effective case discussions. Suggested improvements included increasing the frequency of meetings to allow for the discussion of more cases, improving organization through pre-meeting study of patient cases, implementing a regular oncology board as required by legislation, allocating dedicated and specific time slots for MDTMs, scheduling meetings more than once a week on different days for various specialties, and ensuring the participation of all relevant experts with a clearly defined coordinator role.

Questions concerning the aims and implementation of MDTMs

**Figure 1:**
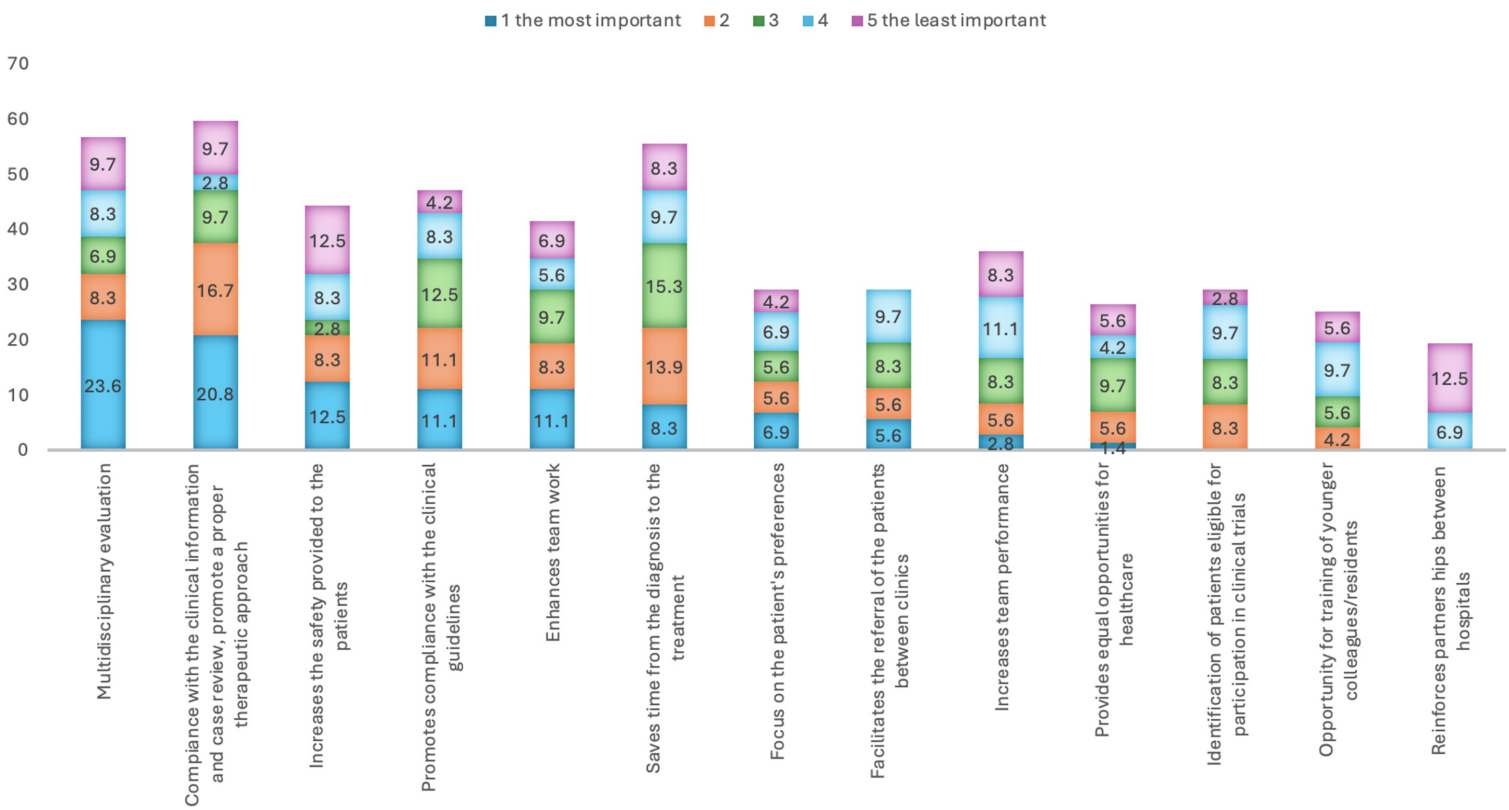
Reasons for conducting MDTMs MDTMs: multidisciplinary team meetings

The most significant reasons for conducting MDTMs are illustrated in Figure 1. The primary purposes identified include multidisciplinary evaluation, alignment with clinical information, and case review, all of which support an appropriate therapeutic approach. Additional benefits highlighted include legal protection and improvements in overall patient prognosis.

Regarding the role of MDTM participants, 51 (70.8%) strongly agree that their role is clear, 55 (76.4%) agree or strongly agree that they engage in discussions, and 84.7% report that their role enhances their professional-cognitive competence.

Questions concerning the structure and guidelines of MDTMs

The majority of participants (n=39, 54.2%) agreed or strongly agreed that MDTM guidelines for patients' medical records and patient information (n=48, 66.6%) are clear, that imaging reports are completed on time (n=29, 40.3%), and that MDTMs effectively support patient management (n=52, 82%). Additionally, they found the meetings efficient given the allocated resources (n=38, 52.8%), beneficial for healthcare professionals' education (n=60, 84.7%), and helpful in developing residents' skills (n=57, 79.2%). Moreover, 35 (48.6%) acknowledged that patients eligible for clinical studies are being monitored. However, most participants only partially agreed that efforts are being made to further develop MDTMs (n=41, 56.9%), that technological support is adequate (n=45, 62.5%), and that histological reports are completed on time (n=22, 30.6%).

**Figure 2:**
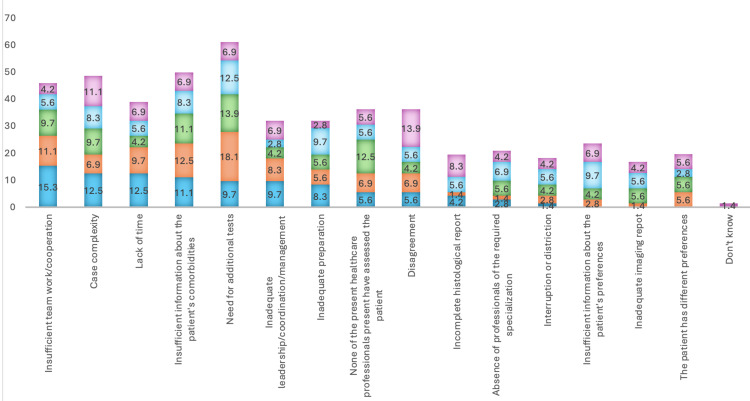
Common obstacles preventing the multidisciplinary team from reaching a treatment recommendation

Participants in MDTMs agreed or strongly agreed that comorbidities (n=67, 93.1%), psychosocial factors (n=59, 82.0%), and patient preferences (n=62, 86.1%) should always be addressed during discussions. Additionally, 48 (66.6%) believed that all oncology patients should be discussed in MDTMs, while 39 (54.1%) felt that some cases should not be discussed in detail. Furthermore, 48 (66.6%) supported the idea that certain patient cases could be managed in mini MDTMs. The main obstacles cited when the multidisciplinary team failed to reach a treatment recommendation were insufficient teamwork or collaboration (n=11, 15.3%), case complexity (n=9, 12.5%), and lack of time (n=9, 12.5%). The obstacles are presented in Table 2 and Figure 2.

**Table 2:** Common obstacles preventing the multidisciplinary team from reaching a treatment recommendation

		Percentage %
1. The most important	Insufficient teamwork/cooperation	15.3
	Case complexity	12.5
	Lack of time	12.5
2.	Need for additional tests	18.1
	Insufficient information about the patient's comorbidities	12.5
3.	None of the present healthcare professionals present have assessed the patient	12.5
4.	Insufficient information about the preferences of the patient	9.7
5. The least important	Disagreement	13.9

Comparisons of questions with demographic and occupational characteristics

Participants aged >45 years agree that the MDTM guidelines for the patient's medical record are clear, the imaging report is completed on time, and psychosocial factors should always be commented on, compared to participants aged <45 years, who moderately agreed.

Residents, specialized physicians, and nursing staff agree that their role in MDTMs is clear and that they actively engage in discussions, unlike secretaries/coordinators, who disagree. Resident doctors and physicians agreed that MDTMs improve clinical skills and that the selection of patients’ eligibility for clinical trials might be further evaluated. In contrast, nursing staff and secretaries/coordinators responded that they did not know. However, all participants agree that certain patient cases can be managed in mini MDTMs, except for secretaries/coordinators, who disagree.

Specialists in pathology/cytology agree that the histological report is completed on time, compared to other specialties, who moderately disagree. Additionally, participants specializing in radiology agreed that the imaging report is completed promptly, compared to those specializing in surgery and medicine, who partially agree.

## Discussion

Healthcare professionals involved in multidisciplinary boards, particularly oncology boards, exhibit a positive approach toward the functioning of these boards while simultaneously identifying areas for improvement. This observation aligns with similar reports from other healthcare systems.

Typically, MDTMs are coordinated by specialized doctors, particularly in the case of oncology patients. Recently, broadening participation by including specialized nurses and specialist coordinators has emerged as one of the primary factors contributing to improvements in the board’s performance.

It is important to note that the number of coordinators, nurses, and specialized doctors who participated in the study was small, indicating challenges in their involvement. The findings suggest that limited availability, the difficulty of arranging staff replacements to cover their clinical duties, and a potential lack of motivation are key barriers to participation. Furthermore, as the study results indicate, medical personnel struggle to balance their workload with the requirements of MDTM participation, which exacerbates inequalities and potentially delays the creation of a comprehensive treatment plan for the patient. Additionally, reports from non-medical staff and resident physicians, though not fully documented in the study due to the small sample size, further corroborate these challenges regarding their limited participation in case discussions.

The interaction between participants, particularly medical and non-medical staff, has also been highlighted in other studies, which report that a purely medical perspective often predominates over the comprehensive treatment plan during MDTMs. Since the role of each healthcare professional in MDTMs is not always clearly defined, establishing specific structures and organizing sessions according to a standardized medical and administrative protocol is essential. Simultaneously, developing the skills of the entire multidisciplinary team is a crucial factor in improving MDTMs. These considerations conclude that coordinators are a prerequisite for the proper functioning of MDTMs and that all doctors and nurses must collaborate closely to achieve this goal.

It should be emphasized that key responsibilities, such as presenting the complete medical history, considering the patient’s psychosocial orientation, outlining rehabilitation methods, and providing additional patient support during hospitalization, as well as incorporating the patient’s preferences or participation in a clinical trial, should be designated to specific healthcare professionals, particularly MDTM nurses [20]. Specifically, oncology board coordinators should organize the patient’s medical record before the individualized discussion at MDTMs [21].

Although many studies have explored incorporating the patient’s views and perspectives into a board’s treatment decision-making, the available data remains limited [22]. Restivo et al. found that psychological, socio-demographic, and other related dimensions were discussed in only 30% of cases. Patient preferences were considered in just 10% of multidisciplinary consultations within the French healthcare system [23]. Divergent treatment priorities between physicians and patients have been observed in several studies, particularly those involving cancer patients. Suppose the primary role of MDTMs is to contribute to an individualized treatment plan and implement the recommendations formulated by the board. In that case, patient values and preferences must be taken seriously. The results of this study indicate that 86.1% of healthcare professionals agree that patient preferences should always be discussed in MDTMs. However, the impact of the patient’s perspective in decision-making could not be fully assessed.

Furthermore, the need to evaluate comorbidities requires further investigation, as only 12.5% of participants in this study considered comorbidities a significant obstacle to decision-making.

Leadership skills and participant interactions are central structural elements throughout the MDTM process. Typically, multidisciplinary team leaders articulate a clear treatment plan [24]. However, fragmented patient information from other board members’ perspectives may influence the discussion, potentially counterbalancing the predominant guidance of a single MDTM member. Therefore, diversity must be considered essential, and any disagreements or alternative treatment plans should be clarified before reaching a unanimous treatment recommendation [25-27].

In modern medicine, particularly oncology, clinical trials are essential for correlating factors and therapeutic interventions to improve survival. In the present study, 48.6% of participants support that MDTMs can play a significant role in this area, yet only 19.4% recognize them as a key tool and one of their main advantages. Efforts to educate multidisciplinary teams to facilitate communication, enhance understanding, and integrate patients into clinical trials have been reported and evaluated in the United Kingdom with encouraging results [28].

MDTMs offer significant advantages, including treatment recommendations based on clinical information, multidisciplinary patient assessments, adherence to clinical guidelines due to the high competence of their members, and enhanced patient safety. However, only 12.5% of participants believe that MDTMs promote adherence to clinical guidelines. Studies suggest that high levels of professional competence and effective teamwork motivate healthcare professionals to refer patients to MDTMs and participate in high-level boards to finalize treatment plans [29]. Additionally, MDTMs are considered valuable for healthcare professional education, with 59.7% acknowledging their benefits, a percentage that increases when moderate responses are included. Referrals to MDTMs are more common in university hospitals than in regional ones (34% vs. 19%), and the impact varies across specialties due to differences in work culture and approaches to teamwork [18].

Obstacles preventing MDTMs from reaching treatment recommendations include insufficient teamwork and collaboration (15.3%), case complexity (12.5%), and lack of time (12.5%). Addressing these challenges is critical, given the rising demand for multidisciplinary boards. Only 30% of participants reported the timely completion of histopathology reports, consistent with findings from other studies. While the absence of the healthcare professional who initially assessed the patient is often highlighted as a barrier internationally, only 12% of participants in this study identified it as a primary concern [2,22]. Additional factors include the need for further tests and insufficient patient history information. Although time constraints are significant, the primary barriers appear to be the lack of leadership, coordination, organization, and teamwork, all of which influence decision-making success. Complex cases contributed to 12.5% of decision-making difficulties, a lower rate than in other studies, likely due to case selection, as university hospitals typically manage more complex cases with greater expertise.

To improve MDTMs, it is imperative to reassess guidelines based on international evidence and ensure the presence of qualified staff tailored to specific conditions, particularly for oncological patients [23,29,30]. Additionally, subgroup presentations within MDTMs, viewed as satisfactory by 70% of participants, are considered an effective method for managing time and resources compared to traditional full MDTMs [29,30].

Reports on developing actions to improve MDTMs' qualitative characteristics are undeniable. All actions aimed at the final implementation of treatment recommendations play a significant role in developing multidisciplinary committees.

Citing the above results is a key element of this study, which seeks to analyze the functioning of healthcare institutions within two major regional health authorities. The breakdown of responses by specialty, hospital type, and the presence or absence of oncology MDTMs enhances the study's relevance. One limitation of this study is the inability to provide a fully tailored questionnaire for different types of MDTMs, as it relied heavily on previous studies with varying qualitative characteristics and significant differences in healthcare systems. Nonetheless, since the Greek national health system has not published encoded protocols for conducting MDTMs, this analysis could further guide developments in this area.

Additionally, the study lacks longitudinal data, which could offer deeper insights into changes in MDTM effectiveness over time. Some self-reported measures may also introduce social desirability bias, as participants might overstate MDTM effectiveness or underreport challenges. Finally, while this study focuses on MDTMs within the Greek national health system, comparisons with MDTM models in other countries (e.g., UK, USA, Australia) are necessary to contextualize the findings and inform potential improvements.

## Conclusions

This study highlights the strengths and challenges of MDTMs within the Greek national health system, emphasizing their crucial role in multidisciplinary decision-making, patient care optimization, and medical education. While healthcare professionals acknowledge the value of MDTMs, key barriers, including time constraints, a lack of leadership structures, and inadequate technological support, limit their effectiveness. To maximize the impact of MDTMs, immediate action should focus on implementing standardized protocols, establishing clear leadership roles, and integrating digital solutions for efficient data sharing. Additionally, investing in structured training programs for MDTM participants can enhance collaboration and improve decision-making efficiency.

Policymakers and hospital administrators must prioritize these improvements to transform MDTMs from an underutilized resource into a central pillar of patient-centered care. Future research should examine the long-term effects of these interventions, particularly their impact on clinical outcomes and healthcare efficiency. By fostering a more structured and collaborative MDTM environment, Greece can progress toward a more integrated and effective multidisciplinary healthcare system.

## Appendices

**Table 3:** MDTM survey questions MDTM: multidisciplinary team meeting

Category	Answer	Response format
Questions about yourself and the time you spend at MDTM		
Sex		Open-ended
Age		Open-ended
Occupation		Open-ended
Specialty		Open-ended
Working Regional Health Authority (YPE)		Open-ended
Number of MDTMs participated/week		Open-ended
MDTM type participated		Open-ended
Time spent on MDTM/week		Open-ended
Opinion on MDTM allocated time		Open-ended
Questions about MDTM purposes and implementation		
What do you consider the most important reasons for MDTM?		Select 5 answers and prioritize (1 = most important, 5 = least important)
Compliance with clinical information/case review promotes a therapeutic approach		
Facilitates referral of patients between clinics		
Interdisciplinary assessment		
Provides equal opportunities for healthcare		
Reduces the time from diagnosis to treatment		
Promotes compliance with clinical guidelines		
Focuses on patient preferences		
Increases team performance		
Enhances teamwork		
Identification of patients eligible to participate in clinical trials		
Opportunity for training of younger colleagues/trainees		
Increases patient safety		
Strengthens collaborations between hospitals		
Are there any other advantages of MDTM that you think are valuable?		Open-ended
My role at MDTM		Use the Likert scale (1-7, 1 = strongly disagree, 7 = strongly agree)
My role in MDTM is clear		
I am involved in the discussions		
My role develops my professional-cognitive ability		
Questions regarding MDTM structure and guidelines		Use the Likert scale (1-7, 1 = strongly disagree, 7 = strongly agree)
MDTM guidelines for the patients’ medical records are clear		
MDTM guidelines for the patients’ information are clear		
We are working for the growth of the MDTM		
The technological support is adequate		
The histological report is completed on time		
The scanning report is completed on time		
The MDTM further supports the management of the patient		
The meetings are efficient based on the resources allocated		
It is beneficial for the education of healthcare professionals		
It develops the skills of the residents		
The patients eligible for clinical studies are being monitored		
Participants’ views on patients discussed in MDTM		Use the Likert scale (1-7, 1 = strongly disagree, 7 = strongly agree)
The comorbidities should always be commented		
The psychosocial factors should always be commented on		
The preferences of patients should always be commented on		
The cases of all oncological patients should be discussed in the MDTM		
Some patient cases should be on the MDTM list but not discussed in detail		
Management of certain patient cases can be done in mini MDTM		
When the MDTM team fails to reach a therapeutic recommendation, what are the most common obstacles?		
Incomplete histological report		
Inadequate imaging report		
Need for additional examinations		Select 5 answers and prioritize (1 = most important, 5 = least important)
Insufficient information on patient comorbidities		
Insufficient information on patient preferences		
The patient has different preferences		
None of the healthcare professionals present has assessed the patient		
Absence of professionals of the required specialty		
Disagreement		
Case complexity		
Insufficient teamwork/cooperation		
Inadequate leadership/coordination/management		
Inadequate preparation		
Lack of time		
Interruption or distraction		
I do not know		

## References

[1] Engelhardt M, Ihorst G, Schumacher M (2021). Multidisciplinary tumor boards and their analyses: the yin and yang of outcome measures. BMC Cancer.

[2] Lamb BW, Brown KF, Nagpal K, Vincent C, Green JS, Sevdalis N (2011). Quality of care management decisions by multidisciplinary cancer teams: a systematic review. Ann Surg Oncol.

[3] Selby P, Popescu R, Lawler M, Butcher H, Costa A (2019). The value and future developments of multidisciplinary team cancer care. Am Soc Clin Oncol Educ Book.

[4] Walraven JE, van der Hel OL, van der Hoeven JJ, Lemmens VE, Verhoeven RH, Desar IM (2022). Factors influencing the quality and functioning of oncological multidisciplinary team meetings: results of a systematic review. BMC Health Serv Res.

[5] Prades J, Remue E, van Hoof E, Borras JM (2015). Is it worth reorganising cancer services on the basis of multidisciplinary teams (MDTs)? A systematic review of the objectives and organisation of MDTs and their impact on patient outcomes. Health Policy.

[6] Pan CC, Kung PT, Wang YH, Chang YC, Wang ST, Tsai WC (2015). Effects of multidisciplinary team care on the survival of patients with different stages of non-small cell lung cancer: a national cohort study. PLoS One.

[7] Munro A, Brown M, Niblock P, Steele R, Carey F (2015). Do Multidisciplinary Team (MDT) processes influence survival in patients with colorectal cancer? A population-based experience. BMC Cancer.

[8] Keating NL, Landrum MB, Lamont EB, Bozeman SR, Shulman LN, McNeil BJ (2013). Tumor boards and the quality of cancer care. J Natl Cancer Inst.

[9] Orgerie MB, Duchange N, Pélicier N (2010). Decision process in oncology: the importance of multidisciplinary meeting (Article in French). Bull Cancer.

[10] Soukup T, Petrides KV, Lamb BW (2016). The anatomy of clinical decision-making in multidisciplinary cancer meetings: a cross-sectional observational study of teams in a natural context. Medicine (Baltimore).

[11] Hong NJ, Wright FC, Gagliardi AR, Paszat LF (2010). Examining the potential relationship between multidisciplinary cancer care and patient survival: an international literature review. J Surg Oncol.

[12] Lamb BW, Wong HW, Vincent C, Green JS, Sevdalis N (2011). Teamwork and team performance in multidisciplinary cancer teams: development and evaluation of an observational assessment tool. BMJ Qual Saf.

[13] Soukup T, Lamb BW, Arora S, Darzi A, Sevdalis N, Green JS (2018). Successful strategies in implementing a multidisciplinary team working in the care of patients with cancer: an overview and synthesis of the available literature. J Multidiscip Healthc.

[14] Lamprell K, Arnolda G, Delaney GP, Liauw W, Braithwaite J (2019). The challenge of putting principles into practice: resource tensions and real-world constraints in multidisciplinary oncology team meetings. Asia Pac J Clin Oncol.

[15] Raine R, Wallace I, Nic a’ Bhaird C (2014). Improving the effectiveness of multidisciplinary team meetings for patients with chronic diseases: a prospective observational study. Health Serv Del Res.

[16] Taylor C, Atkins L, Richardson A, Tarrant R, Ramirez AJ (2012). Measuring the quality of MDT working: an observational approach. BMC Cancer.

[17] Rosell L, Alexandersson N, Hagberg O, Nilbert M (2018). Benefits, barriers and opinions on multidisciplinary team meetings: a survey in Swedish cancer care. BMC Health Serv Res.

[18] Freeman C, Tyrer P (2018). The trainee's guide to research methodology.

[19] Setia MS (2017). Methodology series module 8: designing questionnaires and clinical record forms. Indian J Dermatol.

[20] Punshon G, Endacott R, Aslett P (2017). The experiences of specialist nurses working within the uro-oncology multidisciplinary team in the United Kingdom. Clin Nurse Spec.

[21] Jalil R, Lamb B, Russ S, Sevdalis N, Green JS (2012). The cancer multi-disciplinary team from the coordinators' perspective: results from a national survey in the UK. BMC Health Serv Res.

[22] Lamb BW, Sevdalis N, Arora S, Pinto A, Vincent C, Green JS (2011). Teamwork and team decision-making at multidisciplinary cancer conferences: barriers, facilitators, and opportunities for improvement. World J Surg.

[23] Restivo L, Apostolidis T, Bouhnik AD, Garciaz S, Aurran T, Julian-Reynier C (2016). Patients’ non-medical characteristics contribute to collective medical decision-making at multidisciplinary oncological team meetings. PLoS One.

[24] Hamilton DW, Heaven B, Thomson RG, Wilson JA, Exley C (2016). Multidisciplinary team decision-making in cancer and the absent patient: a qualitative study. BMJ Open.

[25] Rao K, Manya K, Azad A, Lawrentschuk N, Bolton D, Davis ID, Sengupta S (2014). Uro-oncology multidisciplinary meetings at an Australian tertiary referral centre--impact on clinical decision-making and implications for patient inclusion. BJU Int.

[26] Rajan S, Foreman J, Wallis MG, Caldas C, Britton P (2013). Multidisciplinary decisions in breast cancer: does the patient receive what the team has recommended?. Br J Cancer.

[27] Taylor C, Finnegan-John J, Green JS (2014). "No decision about me without me" in the context of cancer multidisciplinary team meetings: a qualitative interview study. BMC Health Serv Res.

[28] Fallowfield L, Langridge C, Jenkins V (2014). Communication skills training for breast cancer teams talking about trials. Breast.

[29] El Saghir NS, Charara RN, Kreidieh FY (2015). Global practice and efficiency of multidisciplinary tumor boards: results of an American Society of Clinical Oncology International survey. J Glob Oncol.

[30] Ryan J, Faragher I (2014). Not all patients need to be discussed in a colorectal cancer MDT meeting. Colorectal Dis.

